# Mastering humanitarianism? A survey of postgraduate humanitarian courses

**DOI:** 10.1007/s10734-021-00797-2

**Published:** 2022-02-04

**Authors:** Adriana A. Stibral, Nazanin Zadeh-Cummings, Matthew Clarke

**Affiliations:** Deakin University, Melbourne Burwood Campus, Burwood, Australia

**Keywords:** Humanitarianism, Postgraduate, Masters, Disasters, Core curriculum, Professionalisation

## Abstract

Humanitarian events are increasing globally, both in number and intensity. In response, the international community spends approximately US$30 billion annually to alleviate both the immediate consequences of these climatic, geographic, and human-induced events but also to support mitigation and recovery. Over the past two decades, the humanitarian sector has increasingly professionalised. One under-studied aspect of this professionalisation is an increase in postgraduate studies in humanitarian action over the last 20 years. Despite this increase, there is no agreement on core curriculum or pedagogy across humanitarian studies courses. How do current Masters of Humanitarian Assistance (MHA) offerings converge and differ, and how can such courses further their contribution to the humanitarian endeavour? This paper surveys 26 anglophone courses offered in the United States, Europe, the United Kingdom, Australia, India, and Nigeria, exploring key characteristics of course entry requirements, flexibility, research, practical components, and academic foci. It does not recommend what a core curriculum for humanitarian courses should be, but does argue that core curriculum for humanitarian courses should be identified by relevant and diverse stakeholders such as affected communities, humanitarian agencies, disaster management bodies, and governments, to ensure that courses in this field provide appropriate learning outcomes. The paper suggests how such a ‘charter’ may be developed.

## Introduction

Humanitarian events are increasing globally, both in number and intensity. Between 2005 and 2015, humanitarian events affected over 1.5 billion people, resulting in 700,000 deaths, 1.4 million injured, and 23 million more losing their homes (UNISDR, 2015). The majority of those affected live within the world’s most vulnerable countries with low international development indicators concerning income, health, education, and human security (Peters, 2017). In response, the international community spends approximately United States (US) $30 billion annually to alleviate both the immediate consequences of these climatic, geographic, and human-induced events but also to support mitigation and recovery (Development Initiatives, 2020: 11). Over the past two decades, the humanitarian sector has increasingly professionalised. This has resulted in the sector, including humanitarian agencies and donors, adopting international standards and best practices. One under-studied aspect of this professionalisation has been an increase in postgraduate studies programs in humanitarian action over the last 20 years.

There are key debates around recognition of humanitarian qualifications—through developing a humanitarian action qualification framework, the introduction of the Humanitarian Passport Project, and humanitarian certifications (Aardema & Churruca Muguruza, 2014; Cranmer et al., 2014). In addition, scholars across various disciplines posit what training and education in humanitarian related fields could or should entail regarding standards, skills, and competencies. Such directions usually target technical areas or sub-specialisations of humanitarian action within academia, such as disaster medicine and crisis or disaster management (Archer & Seynaeve, 2007; Evans et al., 2016; Gallardo et al., 2015; Ingrassia et al., 2014). Despite established debates around the emergence of humanitarianism as a scholarly vocation and academia’s contribution to the humanitarian community (Barnett & Weiss, 2008; Burkle et al., 2009; Burrell Storms et al., 2015), literature on postgraduate-level humanitarian studies or humanitarian action course curricula is limited. Despite the rapid increase of postgraduate humanitarian action programs, there is no agreement on core curriculum or pedagogy across these courses. In other words, there is little understanding or consensus of what it means to hold a Masters in Humanitarian Assistance (MHA). This paper specifically asks how current MHA offerings converge and differ, and how these courses can further their contribution to the humanitarian endeavour.

This paper surveys 26 courses offered in the US, Europe, the United Kingdom (UK), Australia, India, and Nigeria to identify the core components of MHAs to determine what these courses have in common and what makes them distinct. The aim is not to recommend what core curriculum for humanitarian courses should be, but this paper does argue that a core curriculum should be identified by relevant and diverse stakeholders such as affected communities, humanitarian agencies, disaster management bodies, and governments, to ensure that courses in this field provide appropriate learning outcomes. The paper suggests how a charter of core curriculum may be developed.

The following sections provide a background to the topic and describe the approach used to identify the data set of 26 postgraduate courses. Next, a findings section examines commonalities across courses and unpacks how they differ. The discussion section considers the significance of the findings. A penultimate section examines the issue of a core curriculum before the conclusion offers a summary and suggestions for next steps.

## Reforms to humanitarian action and the emergence of humanitarian studies

Humanitarian aid and the humanitarian sector have undergone large-scale changes over the past decades, particularly since the 1990s. Profound failures to deliver humanitarian assistance in a well-coordinated and efficient manner have led to significant reforms and paradigm shifts. New frameworks, standards, and principles have emerged. These include the publication of the first edition of the Sphere Project’s *Humanitarian Charter and Minimum Standards in Disaster Response* (also known as the Sphere Handbook) in 1998, the Core Humanitarian Competencies Framework developed in 2012, and the Core Humanitarian Standard on Quality and Accountability in 2014. Alongside these initiatives aimed at humanitarian practitioners and organisations, 17 donor governments adopted the Principles and Good Practice of Humanitarian Donorship 2003, and 42 donor governments most recently updated the principles to include cash-based programming in 2018. Furthermore, the 2015 Sendai Framework for Disaster Risk and Reduction shifted the focus from disaster response to mitigating risk in order to reducing impacts on communities. All of these initiatives encourage higher levels of technical capacity as requirements for a career in the humanitarian field.

The humanitarian sector is growing financially, responding to protracted crises as well as emergencies, involves a great diversity of actors including those who do not follow mainstream Western approaches, and is becoming increasingly complex. The need for competent, trained humanitarian practitioners is greater than ever before. Sudden, slow-onset, and complex disasters require rapid and efficient responses, mobilising local, national, regional, and international resources and personnel (Edwards, 2009; Sphere, 2020). The number of humanitarian and development aid workers is increasing by approximately 6% annually (Active Learning Network for Accountability and Performance in Humanitarian Action (ALNAP), 2010). An estimated 570,000 field personnel work in the humanitarian sector, with over 500,000 humanitarians being national staff (ALNAP, 2018, 102). While there is an increase of national staff and static numbers or decreasing international staff responding to humanitarian events, expatriate staff remain a significant component of those in leadership roles within the head offices of international aid agencies (ALNAP, 2018) despite calls and commitments in the sector for ‘localisation’ or locally led aid. Employees in the humanitarian field have multiple tasks from raising funds, project design, staff management, and technical roles in health, water, food security, logistics, etc.

Histories of mainstream contemporary international humanitarian action often begin with Henri Dunant’s shocked observations of the Battle of Solferino in 1859 which gave rise to the International Federation of the Red Cross/Crescent movement (Pictet, 1979). However, 100 years earlier, the Great Lisbon Earthquake was just as pivotal in signalling that humanitarian events could be understood less as ‘acts of god’ and more as a consequence of community vulnerability to natural events (Dynes, 2000). As such, not only was it appropriate to respond but it was also appropriate to mitigate such vulnerability and consider social and political actions as causes of vulnerabilities. This recognition is mirrored in contemporary debate around the term ‘natural disasters’—a moniker which obscures human decision-making and its role in disaster impact yet is still prevalent in academic discourse (Chmutina & von Meding, 2019). Furthermore, expressions of humanitarianism and related concepts span both the globe and diverse cultural and religious traditions. The international humanitarian sector, while slowly recognising other concepts and expressions of humanitarianism, is largely rooted in Western concepts and history, as demonstrated by the proclivity to use Dunant and the Battle of Solferino as international humanitarian aid’s origin story.

In the late nineteenth and early twentieth century, cross-border humanitarian responses involved charities established specifically for humanitarian response—primarily related to civilians impacted by war during this time. Across the course of the twentieth century and accelerating following World War II, a transnational humanitarian architecture took shape. At its foundation were four principles derived from the Red Cross’s core principles, dubbed the ‘humanitarian principles’—humanity, independence, impartiality, and neutrality.^1^ Driven by these principles, international humanitarian responses were often amateur in the truest sense of the word—undertaken, led, and funded on an ad hoc basis by those from the Global North with other professional qualifications and primary occupations. In the last three decades of the twentieth century, large-scale humanitarian events captured the attention of the (Northern) public. Media widely reported on human suffering in Biafra (1970s), Ethiopia (1980s), Rwanda (1990s), and Bosnia (1990s). International humanitarian response was also the subject of reporting and attention. This highlighted both the endeavour to address suffering but also the lack of standards, leading the humanitarian sector to pursue greater professionalisation through accreditation, standards, and monitoring and evaluation (Clarke et al., 2019: 2-3). The Sphere Handbook was first published in 1998 to describe the standards required for future humanitarian responses and set out the need for a more professionalised and trained humanitarian workforce. These standards are regularly revised and updated, and in 2019, there were 47,000 downloads of the handbook and over 6,000 people enrolled in Sphere e-learning courses (Sphere, 2020).

Professionalisation of the sector recognises the growing complexity of required responses but also of the wide range of professional roles required within these responses. A recent survey of the sector has identified over 20 discrete professional roles (Bioforce, 2020). Calls for increasing professionalisation have occurred over the last decade (Shanks, 2014; Walker et al., 2010; Walker & Russ, 2011). These calls are indicative of both a reflection of historical failings of the humanitarian sector, but also the evolution of threats to human wellbeing (Kene et al., 2009: 7).

The growing professionalisation of humanitarian aid and the sector is reflected in the rapid expansion of educational initiatives around the world—including training, short courses, and higher education programs such as Masters courses. Across many other fields and disciplines, obtaining a Masters level degree is seen as a requirement for continuing formal education by professional associations, employers, and governments (Drennan & Clarke, 2009). Postgraduate courses require specific curricula and share the commonality of aiming at developing professional practice of students (Armsby et al., 2018). There remain questions as to how humanitarian-specific Masters contribute to professional practice. One study of humanitarian professionals focused on the tension between experience and qualification, finding both a belief that education and professional training is necessary and that qualification inflation is occurring (Clarke et al., 2019). It is the role of formal higher education that this paper is specifically interested in as one aspect of the professionalisation discussion.

There are well-established key debates within the humanitarian sector and academia around the need for a recognition of humanitarian aid-related qualifications, including the introduction of the Humanitarian Passport Project and humanitarian certifications (Aardema & Churruca Muguruza, 2014; Cranmer et al., 2014; Walker et al., 2010). Furthermore, there are a number of solid suggestions and recommendations for the content and design of humanitarian training and higher education related curricula (Aardema & Churruca Muguruza, 2014). However, suggested learning objectives or outcomes are often discipline-specific or technical in their nature. For instance, proposed standards and suggested core knowledge that curricula ought to transmit relate to education in disaster management or disaster medicine, but not specifically to humanitarian studies (Archer & Seynaeve, 2007; Evans et al., 2016; Gallardo et al., 2015; Ingrassia et al., 2014).

Burrell Storms et al. (2015), Burkle et al. (2009), and Barnett and Weiss (2008) discuss and question the role and contribution of humanitarian studies as an academic field to humanitarian work. Walker (2004: 27) examines whether humanitarianism—if deemed being a profession—requires humanitarian academia, meaning the emergence of humanitarianism or humanitarian studies as an academic field of scholarship or discipline in its own right. He argues that academia should bring four key things to humanitarianism: a body of knowledge and research to understand the humanitarian domain, a repository of knowledge, the ability to provide critical and objective advice and finally, and a commonly accepted teaching curriculum that allows for graduating with a recognised formal qualification. Rainhorn et al. (2010) specifically look at higher education initiatives in humanitarian action and provide important figures on humanitarian and related postgraduate courses. Their work dates back 10 years, and course offerings and designs have evolved since as explored in the discussion section. This paper focuses specifically on generalist humanitarian studies Masters, rather than including related courses such as development studies, human rights, and conflict studies. Discipline-specific courses carry the implication of gaining particular knowledge and skills from a known and well-established discipline. This approach differs to that Rainhorn et al. (2010), as well as to that of the International Humanitarian Studies Association on their website directory of postgraduate courses, which both employ a broader lens.

Despite the ongoing professionalisation debate, a gap exists concerning more recent, scholarly literature on the emergence of humanitarian studies as a nascent field of academic scholarship. In this context, scholarly research on the mushrooming of humanitarian action postgraduate university courses is limited. There is no agreement on core curriculum or pedagogy across humanitarian studies courses or consensus of what it means to hold a Masters in this field.

## Approach

The aim of the data collection was to (a) establish the number and location of generalist MHA courses around the world and (b) collate publicly available information on course content and structure for comparison and analysis. The study modified Arksey and O’Malley’s (2005) five-part scoping study framework to arrive at the following approach: (1) defining the research question, (2) identifying relevant programs through database searches, (3) defining and applying the inclusion and exclusion criteria, (4) linked snowballing of included courses for other courses within inclusion criteria, (5) ‘charting’ the data, and (6) reporting the key contrasts and similarities emerging from the charted data.

While Arksey and O’Malley’s (2005) framework was written for literature reviews, this study had similarities with scoping literature reviews—including comprehensive identification of relevant sources (in this case not published studies but Masters program websites) and collation and synthesis of data extracted from relevant sources. Database search (step 2) involved websites that collate Masters programs for prospective students: ‘Find a University Ltd,’ ‘Graduate Prospects Ltd,’ and ‘Studyportal Masters’. Inclusion and exclusion criteria definition (step 3) was an iterative process honed during the database search for relevant programs, rather than fixed before, as it was during the search that the researchers became more familiar with the breadth and depth of the pool of potentially relevant courses. The inclusion criteria were then applied through linked snowballing (step 4), creating a two-step process of data collection consisting of a broader step (database search) and a more targeted step (linked snowballing) designed to identify programs not included in broader step. Charting (step 5), ‘a technique for synthesizing and interpreting qualitative data by sifting, charting, and sorting material according to key issues and themes’ (Arksey & O’Malley, 2005: 26), included collating information on each program’s university, department/faculty/school, country, length, credit units, compulsory and elective subjects, cost, delivery mode, internship options, entry requirements, scholarships, and notable characteristics. Reporting of key similarities and contrasts from the charted data (step 6) has, like Arksey and O’Malley’s (2005) study, an elemental numerical dimension and a categorical organisation. The former refers to the basic information on course numbers and which courses take particular approaches or include particular activities. The latter refers to characteristics of courses—namely course entry requirements, flexibility, research, practical components, and academic foci. These characteristics were developed from reading and charting the data, representing as they do notable points of a program’s identity, as well as areas for convergence and similarity amongst programs.

Inclusion criteria consisted of the following attributes:

- Masters courses (including Master of Arts and Master of Science)
- ‘Humanitarianism’ or ‘Humanitarian Assistance/Aid/Action/Studies/Affairs/Practice’ included in the course title
- Curriculum focus on humanitarian assistance/humanitarian action
- Anglophone courses (coursework, both spoken and written, is predominantly English)
- Courses offered in 2020

These criteria were designed to identify generalist courses, while also recognising the lack of uniform approach to naming such generalist courses. Exclusion criteria included:

- Programs covering some humanitarian aid-related aspects but with different core thematic foci (e.g. Sustainable Development, Peace and Conflict Studies, Emergency Management)
- Short courses, professional certifications, formal training, undergraduate programs, Bachelor’s specialisations, and other postgraduate programs
- Courses whose title includes the term ‘Humanitarian’ followed by a specific field or discipline, such as Management, Logistics, Nursing, Engineering

Programs that included both ‘Humanitarianism’ or ‘Humanitarian Aid/Studies’ or similar and another field in the course title are included. For example, the Masters of Humanitarian and Refugee Studies at University of Maiduguri is included as it includes Humanitarian Studies in the course title, but courses titled Masters of Refugee Studies are excluded. We use the acronym MHA to broadly refer to all programs falling within our inclusion criteria.

The initial database search resulted in a listing of 387 programs offered around the world. Broken down by continent, 276 programs appeared for Europe, 67 for North America, 33 for Oceania, 12 for Asia, 5 for Africa, and one for South America. Further examination of those 387 programs showed that a large number of programs are not specifically humanitarian aid focused and/or titled but include Master courses termed as Humanitarian Engineering, Development Studies, Humanitarian Health Management, International Humanitarian Law and Human Rights, Humanitarian Logistics, Risk and Disaster Science, Disaster Management, and Peacebuilding and Law, amongst others. A total of 26 courses emerged as satisfying the inclusion criteria (see Table 1). All courses except three (Masters of Humanitarian and Refugee Studies, University of Maiduguri, Nigeria; Professional Master of Humanitarian and Refugee Studies, University of Ibadan, Nigeria; and Master of Science in Compassion, Peace, Humanitarian Action, and Disaster Risk Management, MIT World Peace University, India) are offered by institutions located in the Global North.

**Table 1:** Courses reviewed

Title	University	Country or region
MSc Humanitarian Action	University of London	United Kingdom
MA in Humanitarian Action and Peacebuilding	United Nations Institute for Training and Research (UNITAR) and Oxford Brookes University	Switzerland and United Kingdom
MSc Humanitarianism, Conflict and Development	University of Bath	United Kingdom
MSc International Development and Humanitarian Emergencies	London School of Economics	United Kingdom
MSc Humanitarian Practice	University of Manchester	United Kingdom
MA Humanitarianism and Conflict Response	University of Manchester	United Kingdom
MSc International Disaster Management and Humanitarian Response	University of Manchester	United Kingdom
MSc International Humanitarian Affairs	University of York	United Kingdom
MSc Humanitarian Studies	Liverpool School of Tropical Medicine	United Kingdom
Erasmus Mundus Joint Masters Programme in International Humanitarian Action	Network on Humanitarian Action (NOHA) member universities^a^	Europe
Master of Arts in Humanitarian Action	University of Malta	Malta
MSc Humanitarian Action	University College Dublin	Ireland
Masters in Humanitarian Action and Conflict	Uppsala Universitet	Sweden
Master in Humanitarian Action, Cooperation and Development	Universidade Fernando Pessoa	Portugal
MA in Human Rights and Humanitarian Action	Sciences Po	France
Master of Advanced Studies in Humanitarian Action	Geneva Centre of Humanitarian Studies	Switzerland
MA Humanitarian Assistance and Crisis Management	School of International Training	United States of America
MSc Humanitarian Studies	Fordham University	United States of America
MA in International Humanitarian Action	Fordham University	United States of America
MA in Humanitarian Assistance	Tufts University	United Sates of America
Master of Humanitarian Assistance	Deakin University	Australia
Master of Sustainable Development and Humanitarian Action	Deakin University	Australia
Master of Humanitarian and Development Studies	Western Sydney University	Australia
Masters of Humanitarian and Refugee Studies	University of Maiduguri	Nigeria
Professional Master of Humanitarian and Refugee Studies	University of Ibadan	Nigeria
Master of Science Compassion, Peace, Humanitarian Action & Disaster Risk Management	MIT World Peace University	India

^a^These include Aix-Marseille Université (France), University of Malta (Malta), Rijksuniversiteit Groningen (Netherlands), Ruhr-Universität Bochum (Germany), Universidad de Deusto (Spain), University College Dublin (Ireland), Uniwersytet Warszawski (Poland), Uppsala Universitet (Sweden), and Vilniaus Universitetas (Lithuania). Three of these universities (Malta, Dublin, and Uppsala) offer an additional Master’s program relevant to this study, which are included separate to the NOHA program.

This study’s data set is built on publicly available sources. The limitations of this approach include a lack of qualitative data from methods such as surveys, no access to student feedback on courses or their employment outcomes following graduation, and a lack of data on staff approaches to pedagogy. These data sources could form the basis for future, complementary studies. The focus of this paper is on the course structure and central course components, including key subjects and activities that form part of the curriculum, making the available data sufficient for the inquiry.

## Findings

The findings are grouped into five course characteristics: course entry requirements, flexibility, research, practical components, and academic foci (e.g. core themes in the curriculum and subjects taught). This section first looks at the commonalities in these categories and then at the convergences.

### Key commonalities and similarities

It was possible to identify aspects that were common across the 26 postgraduate courses surveyed as part of this review. These similarities indicate there may be sufficient common practice to identify a tentative core approach and curriculum. However, the skewing of the courses to universities in the Global North brings a hazard that a common curriculum determined solely by the universities offering courses would neglect perspectives from the Global South. These commonalities serve as a basis for understanding the current state and the potential of a common curriculum, but do not in and of themselves constitute a proposed common curriculum.

Course entry requirements are similar across all MHA courses: the successful completion of an undergraduate in the same or similar field; some programs required applicants to submit a formal application that includes a curriculum vitae, a letter of motivation, and/or letters of references; and where English is not the applicant’s native language, an International English Language Testing System (IELTS) test score of 6.5 or 7.0 is required. Out of 26 programs, ten either mentioned that professional experience is preferable and/or permit applicants without a Bachelor’s degree if they hold 2 to 5 years relevant professional experience. Fourteen out of 26 programs did not mention the relevance or need for previous practical experience. A strict requirement of having 2 to 5 years of professional experience in the humanitarian sector in order to successfully apply for course entry was the case for the remaining four out of the analysed 26 programs.

Flexibility is a major aspect of all 26 MHA courses. Categories of flexibility include the length of the program, delivery mode, and location. The majority of courses are either one or 2 years full-time. Nearly all programs offer flexibility in studying part-time, full-time, or a combination of both. This, in turn, impacts the length of the program, depending on the modality of study a student chooses. Various programs also included options to complete intensives and on-campus blocks, including overseas. Sixteen out of 26 programs offer most or all components of their curricula online, while five MHA degree programs are delivered exclusively online. Ten programs are seemingly offered only on-campus. Eleven programs are delivered in a blended format (online and on-campus)—many of which include a residential or overseas intensive component. The modality of studying is connected to location flexibility. Multiple programs show flexibility in locations where students attend classes and complete other course requirements such as research, fieldwork, placements, and internships. For example, the Network on Humanitarian Assistance (NOHA) program is offered by eight European universities and allows students to choose the desired location for their compulsory semester abroad at one of NOHA’s partner universities.

Research is a core requirement for all Master of Humanitarian Action programs. The majority of programs (19) require students to complete a Masters dissertation/thesis. In the seven programs where a dissertation is not required, students must complete a research-related capstone, undertake a research project, and/or submit a research paper. The majority of programs also embed compulsory research-related seminars, capstone units, workshops, or subjects (e.g. Research Methods, Research and Ethics, Research Project).

Practical components are optional or compulsory in approximately half of the 26 MHA programs. Fourteen programs include a mandatory practical component in form of a placement, practicum, fieldwork, simulation-based unit, training, or an internship. One program offers practical components as an elective. Eleven out of 26 MHA programs have no practical component in form of a placement, internship, training, simulation-based learning, or fieldwork as a requirement for course completion. Practical components vary across universities with regard to the type of practice-based learning. For example, the NOHA program requires students to undertake ‘regional training’ at a partner university and the completion of an internship placement. Western Sydney University and Sciences Po require students to complete either an internship or an overseas study exchange. The School of International Training embeds a mandatory field practicum in Jordan, Switzerland, or Uganda. The University of Maiduguri and the University of Ibadan encourage students to complete their compulsory internship in a refugee or internally displaced people (IDP) camp in Africa. Where a professional practice component is not a mandatory requirement, students usually are provided with the opportunity to complete fieldwork or a placement as an elective subject/unit.

Academic foci, themes, and subject areas that MHA programs cover in their curricula (to a varying extent) include history of humanitarianism, humanitarian principles and frameworks, aid in theory and practice, key issues in humanitarian (and development) practice, critique of humanitarian aid practice, conflict and security, peacebuilding, (international) development (aid), sustainable development, politics/global governance, global/public health, human rights, forced migration, refugees, displacement, politics and globalisation, reconstruction and re-building, technical sectors in humanitarian aid response, media, advocacy and communication, geographic foci (e.g. Asia, Middle East, Africa), leadership, teamwork, self-management, fundamentals of research, research dissertation, placement/practicum, training, and fieldwork. This is a broad church and demonstrates the lack of consensus around what knowledge an MHA should signify. It also shows the vastness of fields connected to the humanitarian realm.

Common themes, subject areas, research, and practical components that are comprehensively integrated by the majority of all 26 analysed MHA programs in their curricula and thus could qualify as common principal curriculum components are listed in Table 2.

**Table 2:** Core themes, topics, and subject areas

Theme or subject area	Number of programs where this is a core component in the curriculum	Number of programs where this is an optional component (i.e. elective unit) in the curriculum
Research and dissertation	26	N/A
History of humanitarianism and the humanitarian system—principles, frameworks, ethics, cutting issues in development, and aid practice	19	2
Disaster and emergency management	8	6
Internship/placement/practicum	14	1
Global governance, international relations, world politics, complex humanitarian emergencies, the political economy of aid	11	9
Management	13	3
Global/public health	10	7
Development/development studies/sustainable development	9	5
Conflict, conflict resolution, peace, and peacebuilding	8	7
Protection and international humanitarian law	9	2
Leadership, self-management, teamwork	4	N/A
Human rights	3	5
Technical/sector-related aspects of humanitarian response	3	1
Negotiation and diplomacy	2	5
Gender/gender-based violence/feminism	2	4
Media, advocacy, and communication	1	6

However, construction of a core curriculum must use such data and analysis only as one component in its final construction. The table above quantifies and demonstrates trends as they are, but does not make normative judgements on whether these areas are what MHA programs should be focusing on. There may be areas missing from the current trends that would serve a core curriculum well. For example, the majority of programs do not appear to focus on critical examination or critique of the humanitarian sector, though it does seem to be an emerging theme in some programs.

### Key differences

There were notable distinctions within the surveyed courses. Such aspects demonstrate the need for courses to provide options for students and to develop specialisations and areas of expertise. They also demonstrate how universities themselves differ in terms of their own approach to pedagogy.

Some programs have very specific academic foci. Some courses place the main emphasis on technical or other, quite particular aspects linked to humanitarian assistance. Those, for example, include food security/food and nutrition in emergencies (e.g. University of York, University of London, Deakin University, and Tufts University), climate change (University of London, University of Manchester, Liverpool School of Tropical Medicine, and Geneva Centre of Humanitarian Studies), or logistics and supply management (Liverpool School of Tropical Medicine and Fordham University). Emerging non-traditional themes and subject areas that some programs offer as elective or core units also include anthropology (University of Manchester, NOHA, and Sciences Po), human(itarian) resource planning and administration (University of Manchester and Fordham University). Thus, even ‘generalist’ humanitarian degrees often focus on particular aspects of humanitarianism, making them more specialised than their titles may reveal.

Other identified differences in the analysed 26 MHA programs include a varying level of interdisciplinarity. Some programs stressed the importance of interdisciplinary teaching and research, whereas other programs had a quite discipline-specific focus. MHA programs are housed in various departments, faculties, and schools. They include Health Science, Law and Political Science, International Relations, Human Rights, and Theology. This also suggests that programs approach their research components from a variety of methodologies depending on the discipline in which they sit.

Furthermore, programs offered by universities in the USA, Australia, and the UK notably offered various exit options for students who decide not to complete the entire Masters program. Options include Graduate Diplomas, Graduate Certificates, Postgraduate Diplomas, and Postgraduate Certificates. Some programs target a broader-level audience, while other programs are specifically aimed at practitioners already working in the humanitarian aid sector.

## Discussion

Before turning to discussion of the course component analysis, this section considers two important points: course prevalence and Global North dominance. Rainhorn et al. (2010) identified 39 humanitarian Masters programs. These included programs in English, French, and Spanish, as well as courses whose titles did not include the word ‘humanitarian’, such as Masters in Post-war Recovery Studies, and courses that included humanitarian studies as a concentration within a wider Masters, such as Masters of Science Food Policy and Applied Nutrition with Specialisation in Humanitarian Assistance. Applying our selection criteria to their data set, the number of programs dwindles to ten.^2^ This includes four NOHA degrees counted separately per university—as they are one program, we count them only once in total. Thus, the comparable number is seven programs. This suggests nearly a quadrupling of generalist anglophone humanitarian masters courses in 10 years.

One important aspect to note is the overwhelming presence of universities in the Global North in the data set. Two Nigerian universities and one in India offer courses that fell within the inclusion criteria, but otherwise all universities are located in the US, Europe, the UK, or Australia. The anglophone inclusion criteria is one limiting factor—courses such as Université Catholique d’Afrique Centrale’s (Cameroon) Master en Droits de l’homme et Action humanitaire (Master in Human Rights and Humanitarian Action) are excluded. However, it is also important to recognise that many universities across the world offer English instruction—mirroring one aspect of the neo-colonial influence on the tertiary sector that sees anglophone research valued and legitimised over knowledge in other languages (Suzina, 2021). Our data collection method, which relied on publicly available information accessible online, may also have omitted courses that do not have an online presence. There is some web suggestion, for example, of a Masters Degree in Humanitarian and Conflict Studies from the University of Juba (South Sudan), but no accessible university, course, or other website to confirm or from which to draw data on the course.

Global power dynamics affecting tertiary education more broadly impact humanitarian studies programs, where Global North-based universities often have more access to resources, travel, and reputational clout than their counterparts in the Global South. Simultaneously, universities in the Global South are pressed by neo-colonial structures—as Ndlovu-Gatsheni writes, ‘universities in Africa are sites for a reproduction of coloniality’ (Ndlovu-Gatsheni, 2020: 35). In North-South research collaborations, for example, Ishengoma finds enduring ‘relations of paternalism and privilege’ (Ishengoma, 2016: 153). In the humanitarian studies sector, these patterns of power, privilege, and paternalism manifest in a phenomenon where Global North universities educate (and often receive tuition fees from) students for careers that may have them living in and working in the Global South. These students may themselves be from the Global South and migrate to the Global North for study. Some programs in the Global North offer or require students to complete coursework at universities in the Global South, but ultimately the degree-granting institutions are those based in the Global North. Simultaneously, the mainstream international humanitarian sector is grappling with issues of colonialism, power imbalances, and issues of which actors have access to resources for response (Gómez, 2021; Jayawickrama, 2018; Shifting the Power, 2017). Humanitarian studies masters, which often occupy the dual track of unjust Global North power dominance from both the humanitarian and higher education world, can at worst be seen as tools for replicating and perpetuating these imbalances. This suggests a crucial role for critical perspectives, innovative research in true partnership with Global South counterparts, and reflection in and amongst Global North MHA programs on the power they hold.

The trends in course entry requirements indicate a range of understanding of what is required to be a successful Masters candidate in this field. A quarter of courses permit students without a Bachelor’s degree to enrol, indicating some recognition of professional experience as equivalent to undergraduate study but not a unified position on this. Only four courses strictly require professional experience. This suggests a minority view of humanitarian studies courses as having a primary function of building on professional experience—despite previous findings that mid-career study can provide valuable space for thinking and reflection (Clarke et al., 2019). Instead, entry requirements more broadly suggest that both students who have not yet begun careers in the sector and those already working in the humanitarian realm are seen as potentially successful candidates. Humanitarian studies programs thus appear to play the dual role of preparing new humanitarian professionals and deepening the knowledge base of individuals already in the sector. MHA graduates lacking any practical experience may not be merely naïve or underprepared, but even ‘dangerous’ due to ‘over confidence and over application of book learning’ (Walker & Russ, 2010: 47). The role of practical experience within programs, from this perspective, is thus not only a benefit to the student but necessary to avoid causing undue harm.

Even before COVID-19, flexible course modalities were becoming an increasingly popular option. It is a difficult moment to understand the long-term impacts of COVID-19-induced modality flexibility. Quick shifts to online learning due to the pandemic have obfuscated the potential for impactful online learning as these changes were done in time-pressured, stressful, emergency circumstances (Watermeyer et al., 2021). The data for this paper was collected in late 2019 and early 2020, capturing the prevalence of location and modality flexibility before and early in the pandemic. The characteristics of location and delivery flexibility suggest that flexible modalities will continue in MHA programs even with increased opportunities for travel and in-person study.

Online courses certainly have some access benefits. Students do not need to relocate, which allows them greater flexibility to maintain all or some of their job and family commitments. International online students typically do not need visas and can study at institutions with desirable programs but without potentially unaffordable living and relocation costs that come with physical attendance (Watermeyer et al., 2021: 629). Asynchronous online courses allow students to study in their own time, furthering their ability to fit study into their professional and personal lives—though the need for flexibility can sometimes translate into poorer performance from online students compared to their in-person peers (McParlan et al., 2021).

Yet online study is by no means ‘accessible’ in the broadest sense of the word and should not be viewed as a panacea to the Global North/Global South imbalance in MHA programs. Tuition fees can be at best a carefully considered investment for students with well-paying salaries or savings in the Global North, and at worst wholly prohibitive, particularly for would-be students earning and saving in the Global South. Potential students without reliable access to the internet, at a quality that can support high-bandwidth activities such as lecture recordings, downloading research papers, and uploading assessments, do not benefit from the availability of online courses. Furthermore, ‘access’ to online learning does not necessarily translate into success in or uptake of online learning, with other variables influencing student success and enrolment in flexible or entirely online learning modes. For example, previous research has found gendered dimensions to self-perceived capacity to succeed in online learning (Shen et al., 2013), and that students can choose in-person learning—even if more notably more inconvenient in terms of resources—over comparable online learning if they believe the in-person environment to be more optimal (O’Neill & Sai, 2014). This questions the ability of even flexible modality MHA programs to reach students that in-person programs, particularly those based in high cost of living areas, cannot.

Hammond (2019: 2) highlights that research has ‘direct relevance to the needs of various sectors, scholarship and the generation of ideas’. The propensity of research in MHA programs suggests that research skills are broadly recognised as central to earning a humanitarian Masters. This distinguishes accredited postgraduate degrees from other forms of humanitarian education, such as short courses, which may or may not have a research component. However, the source of the recognition is unclear—whether both tertiary institutions and humanitarian stakeholders such as affected populations, agencies, donors, and governments all equally value research skills is unknown. Additionally, the extent of the transferability of academic research skills to related humanitarian work, such as monitoring, evaluation, and learning, is unclear. Evaluation and research, while linked, are distinct (Levin-Rozalis, 2003). While research may be a common recognised component of Masters level programs, further work must be done to validate if postgraduate research skills are fit for purpose for humanitarian careers. The widely varied disciplinary homes of MHA programs—and thus wide variety of research methods taught and employed—further questions how these skills translate across programs as a whole.

## A process towards a charter of core curriculum

Over a decade ago, Walker and Russ (2010) highlighted that a lack of an agreed core curriculum prevents potential employers from having a solid understanding of the skills and knowledge possessed by an MHA graduate. Additionally, their study found that MHA programs ‘were being offered without much of an understanding for what the industry needed in terms of numbers and skills’ (47). This paper proposes value in a core curriculum not only to benefit hiring managers in international humanitarian agencies, but also as a tool for incorporating voices of other key stakeholders including affected populations, local organisations, disaster management bodies, and governments. In this sense, what this paper proposes goes beyond a competency mismatch evaluation, a form of assessment between what an education provides versus what employers expect (Peng et al., 2016). Humanitarian education must go beyond competency checks of the sector as it exists in the eyes of employers, but also look at skills and qualities needed for the sector to positively transform through a wider set of viewpoints.

It is insufficient for those teaching humanitarian education to solely determine what core curriculum needs to be. Given the impact this work has on communities, it is necessary for all curricula to be evidence-based, draw heavily on lived experience, and be informed by industry needs and gaps. Multiple stakeholders are therefore necessary to identify what core curriculum is required. In addition to academics, relevant stakeholders will include humanitarian aid agencies, donors, governments, multilateral humanitarian agencies, and communities themselves that are impacted by humanitarian events. It is important that stakeholders represent the wider experience of the humanitarian sector and that stakeholders from the Global South are not excluded. Failure to do so will result in a core curriculum that will lack the depth and width required.

Given the increasing requirement for postgraduate qualifications within this sector—both as a pre-requisite requirement for hiring but more so in recognition of the increasing skills necessary for a complicated, integrated global industry—it may be that a base core curriculum could be agreed upon by key stakeholders to ensure minimum education standards are achieved and graduates are prepared for ethical, impactful, and changemaking careers in the humanitarian sector. Such core curriculum would provide a basis for assessing entry-level competency and building of specialist knowledge. Such international accreditation exists for example in discipline such as business (such as AACSB International) and engineering (such as the Washington Accord).

There have been previous studies and efforts to move towards a core curriculum. Clarke et al. (2019) suggest curriculum that should be included in postgraduate qualifications in humanitarian studies. This includes scaffolded and integrated international experience, soft skills (including inter-cultural communication, teamwork, and safe work habits), language acquisition, and technical skills in monitoring and evaluation. There is also recommendation that curricula be differentiated if students were undertaking postgraduate study at the commencement of their humanitarian career or during a mid-career break. NOHA is a consortium of universities that in some regard have identified a common curriculum, requiring students to complete postgraduate studies in humanitarian action across various member universities. Since the mid-1990s, NOHA has effectively accredited the curriculum within members’ courses to facilitate this provision of credit from different institutions towards a single degree. From this common base, students can seek the specialisations available in different institutions. However, the NOHA program is limited to its degree-granting members, all of which are based in Europe. The European Universities on Professionalisation on Humanitarian Action (EUPRHA) program was a NOHA initiative involving thirty European universities, NOHA alumni, and two international humanitarian organisations. EUPRHA aimed to create a humanitarian studies qualifications framework within the Bologna Declaration structure for European universities, as well as map humanitarian assistance in Europe (Soitu, 2014).

NOHA/EUPRHA provide insights into how a wider ‘charter’ or ‘declaration’ on core curriculum for postgraduate humanitarian courses in humanitarian action might be designed. As discussed above, a process of consultation must include a wide range of stakeholders for it to adequately capture the wide needs of the humanitarian sector. Key stakeholders must include universities themselves, but must also include a human resource representatives from large and small aid agencies, civil society groups, specialist aid agencies (in terms of sectoral specialisation), and donors. The viewpoints of people impacted by humanitarian crisis should be heard—both through some of the aforementioned stakeholders and also as stakeholders in their own right. Through various rounds of consultation, a core curriculum may be identified that provides a satisfactory basis for study within humanitarian action that meets not only the current needs of the sector and provides base capability for employing agencies, but further equips graduates to instigate positive changes in the sector. Successful completion of such core curriculum would warrant basic knowledge and skills have been obtained. To gain currency, stakeholders would have to value this core curriculum and preference applicants with such qualifications.

Given academic independence, it is expected that a charter of core curriculum would be voluntary. There is no expectation that core curriculum could be made mandatory nor that would it be licenced. Rather, participating universities would voluntarily commit to this curriculum. In time, it might be envisaged that an accreditation body could be established to assess adherence to this core curriculum in similar vein to accreditation of Masters of Business Administration. Within this approach, such accreditation may result in a ‘gold standard’ for postgraduate courses in this field that will be understood in the sector. Such a code could also positively act to rebalance the bias that is currently evident in the predominance of humanitarian courses being located in Global North universities. Such a code would enhance the credibility of a course regardless of where it is taught (Global South or Global North) or by which mode (face to face, online, mixed mode). A core curriculum minimises the distinction between universities through a level of assurance that adherence to this voluntary code would give to both students and prospective employers in the sector. In this way, the teaching of humanitarian studies could be localised and democratised further than it is currently.

## Conclusion

Despite debates in the humanitarian sector around professionalisation and reforms that aimed to make response more effective, there is little understanding of what holding a generalist MHA signifies. How do MHA offerings converge, and how do they differ? Furthermore, how can these courses further contribute to an improved humanitarian sector? Twenty-six MHA programs from around the world were analysed as part of this MHA course survey. The prescribed key selection criteria for identifying and analysing MHA programs include anglophone programs and ‘Humanitarian Action/Assistance/Aid’ (or similar) to appear in the course title and present a core focus of the curricula.

The majority of such MHA programs are offered in the Global North, with two degrees offered in Nigerian universities and one in India. Institutions located in other regions of the world offer additional Master-level programs in similar or allied fields such as development and disaster management, however not specifically titled as an MHA. This presents the first implication of this research: the need for further unpacking how humanitarian studies relate to these allied fields. If allied fields like development studies, forced migration and refugee studies, and emergency and disaster management are seen as part of or equivalent to humanitarian studies, then the discussion surrounding a core curriculum must be broadened to those fields and respective stakeholders.

This study found notable areas of commonality and divergence amongst MHA programs to include course entry requirements, flexibility, research, practical components, and academic foci. A second implication concerns pedagogy. This study revealed the wide range of academic themes and disciplinary ‘homes’ found in MHA programs. It is worthwhile to note that some themes and topics (e.g. public health and management) are rather emerging common themes than subject areas shared by all or the majority of all 26 MHA programs. The critical examination of humanitarian aid and critique of the sector also seem to be only an emerging impetus. Further research could entail the comparison and analysis of MHA course learning outcomes, core units/subjects, assessment, teaching and learning pedagogies, staff composition, university-industry partnerships, student satisfaction, and graduate employability.

Acknowledging the diversity within and between different MHA programs, there is much room for differences in approach and in the belief of what constitutes ‘core’ or ‘common’ concepts. A third implication of the research is the potential for a common curriculum. To ensure these postgraduate courses best support the humanitarian endeavour, consideration should be given to the development of core curriculum that sets the basis for knowledge and capabilities required by the increasingly complicated humanitarian industry. Such a charter would be voluntary and result from wide consultation from across key stakeholders in this sector. Given the numbers of people affected annually and the likelihood of such events increasing in quantity and intensity, it is important that educational outcomes can be warranted as serving the needs of affected populations.

### Availability of data and material

Not applicable

### Code availability

Not applicable

## Footnotes

1 Three additional principles are included in the Red Cross’s fundamental principles: universality, unity, and voluntary service.

2 Of these ten courses, four were included in one entry under the Network on Humanitarian Action, one is no longer offered (Master of International Humanitarian and Social Work at Palacky University, Czech Republic), and the remaining five are included in our data set.
